# Analysis of the Energy Expenditure of Sports School Activities in Children

**DOI:** 10.3390/children11080946

**Published:** 2024-08-05

**Authors:** Daniel González-Devesa, Miguel Adriano Sanchez-Lastra, Carlos Ayán-Pérez, Nerea Blanco-Martínez, María Soutullo Rivas, María Vidal-Mariño, Silvia Varela

**Affiliations:** 1 Well-Move Research Group, Galicia Sur Health Research Institute (IIS Galicia Sur), SERGAS-UVIGO, 36310 Vigo, Spainmisanchez@uvigo.gal (M.A.S.-L.); silviavm@uvigo.gal (S.V.); 2 Departamento de Didácticas Especiáis, Universidade de Vigo, 36310 Vigo, Spain; 3 Facultad de Ciencias de la Educación y del Deporte, Universidad de Vigo, Campus a Xunqueira, s/n, 36005 Pontevedra, Spain; nereablancomartinez@gmail.com (N.B.-M.); msoutullo14@gmail.com (M.S.R.); mariavidalmarino@gmail.com (M.V.-M.)

**Keywords:** child health, physical fitness, aerobic endurance, sport program, human development, metabolic equivalent

## Abstract

(1) Background: This study explores the potential energy expenditure associated with participation in after-school sports activities among primary school children. (2) Methods: The study involved 129 children age (11.35 ± 0.55 years) recruited from eight different public after-school sport programs. (3) Results: Data analyses revealed significant differences between the eight sports in total calories per session, calories per minute, and METs (*p* < 0.05). All sports showed higher energy expenditure compared to chess (*p* < 0.05), with soccer and rugby exhibiting the highest energy expenditure per session. Team sports showed elevated energy consumption per session (*p* < 0.01, r > 0.30), calories per minute (*p* = 0.01, r > 0.40), and METs (*p* < 0.01, r > 0.40) in comparison with individual sports. (4) Conclusions: These findings enhance our understanding of the energy expenditure observed in primary school children following various after-school sports activities. The results indicate that team sports, in particular, are pivotal in elevating physical activity levels, thereby playing an essential role in fostering healthier lifestyles among children.

## 1. Introduction

Childhood obesity and sedentary lifestyles have become major public health problems that pose significant challenges to the wellbeing of children and adolescents around the world [1]. The causes of childhood obesity are multifaceted, involving a complex interplay of genetic, environmental, and behavioral factors. The ramifications extend beyond physical health, as research indicates that obesity also exerts a detrimental influence on the quality of life among children [2]. Moreover, having obese status from childhood may place children at increased risk of becoming obese adults [3]. This problem is further accentuated by the array of chronic diseases correlated with obesity in adulthood, encompassing conditions such as hypertension [4], cardiovascular disease [5], and diabetes [6].

One of the strategies that could help improve children’s health is the promotion of physical exercise [7]. Regular physical exercise was demonstrated to be effective at controlling blood pressure, lipid profile, metabolic syndrome, overweight, and obesity in children and youth [8,9]. Moreover, physical activity has demonstrated benefits beyond physical health; previous research indicates its positive effects on executive functions, attention [10], and academic performance [11] among children and adolescents.

Engaging in physical activity from an early age is the most effective method for the primary prevention of obesity. This approach helps establish a positive relationship and attitude towards physical activity, encouraging its lifelong practice [12].

It is considered that a child is sufficiently active and can achieve the aforementioned benefits when reaching the physical activity levels recommended by the World Health Organization (WHO) [13]. This recommendation specifies that children aged 5 to 17 should engage in at least 60 min daily of moderate to vigorous physical activities, primarily aerobic, and include vigorous activities at least 3 days a week. WHO defines moderate-intensity physical activities as those achieving an energy expenditure of between 3 and 6 metabolic equivalents (METs), which is 3 to 6 times higher than the energy expenditure at rest (1 MET). Vigorous activities are those exceeding 6 METs. Therefore, children should reach more than 180 MET-minutes per day of moderate- to vigorous-intensity physical activity. Additionally, these guidelines indicate that children and adolescents should limit sedentary time, particularly screen time, during leisure. Sedentary behavior is defined as any waking behavior characterized by an energy expenditure equal to or less than 1.5 METs, whether in seated, reclined, or lying positions.

Despite the numerous benefits associated with attaining adequate levels of physical activity and the detrimental consequences of falling short, research indicates that a significant proportion of adolescents aged 11 to 17 worldwide, estimated at 81%, do not meet recommended activity levels [14]. A systematic review conducted by Tomkinson et al. [15] highlighted a decline in cardiorespiratory fitness levels among children and adolescents over the past three decades, a factor identified as critical by the WHO for this demographic [16]. Consequently, there exists an urgent imperative to expand upon established effective policies and programs aimed at boosting physical activity levels in the population, with a particular focus on children and adolescents [14].

Due to the recognized importance of physical activity in children’s development and overall health, extensive research has been conducted to understand the energy expenditure in various activities. Ridley, Ainsworth, and Olds [17] developed the Compendium of Energy Expenditures for youth, and some authors have tried to expand on this information [18]. In these regard, previous studies have been focused on energy expenditure, during summer months [19], school recess [20], transport to school [21], and physical education classes [22].

Of growing interest is the role that after-school time plays in the energy expenditure of the children [23]. Several studies have been conducted, aimed at designing interventions applicable to after-school hours, which do not appear to have a significant impact on increasing physical activity levels [24,25]. However, fewer studies have been conducted on existing extracurricular activities. This is important, as an after-school intervention could increase daily children’s energy expenditure [26] as well as promote the development of motor skills and abilities at an early age [27]. Participation in after-school sports programs could increase children’s physical capacity and physical activity levels [28]. Therefore, it could be a valid option for children to get started in sport and follow active lifestyles.

In Spain, 65% of schools organize some type of sports or physical activity at least once a week after school hours. Approximately 72% of girls and 75% of boys are enrolled in these activities [29]. For extracurricular activities to substantially contribute to meeting international physical activity recommendations, it is necessary that they are performed at appropriate intensity levels (at least 3 METs) throughout the duration of the sessions.

Therefore, it is necessary to further investigate the measurement of children’s energy expenditure in after-school sports activities. This information could be of interest to parents, guardians, teachers, and professionals in order to establish effective physical activity strategies for children. Consequently, this study aimed to investigate the energy expenditure of primary school children in sports activities after school hours.

## 2. Materials and Methods

### 2.1. Participants

This cross-sectional study involved healthy urban children who were selected from eight distinct public after-school sports programs located in the northern part of Spain. The data collection was conducted between November 2021 and January 2022. The public after-school sports programs were located in one municipality, with a population size of approximately 85,000 inhabitants. Due to the explorative character of the study, no sample size calculation was conducted.

The criteria for inclusion required the children to be aged between 10 and 12 years, actively participate in after-school sports at least twice per week, able to understand Spanish, and not suffer from any health conditions that could negatively affect their participation in the research activities.

Exclusion criteria were set for children who had intellectual or physical disabilities that could prevent them from fully understanding the testing protocols or from performing the tests accurately.

The participants were free to choose the sporting activity in which they would participate. Written informed consent was received from the parents or guardians of all the children who took part in the research study. Participants were not provided with monetary compensation for their participation. The study was registered on Open Science Framework (OSF, https://doi.org/10.17605/OSF.IO/FGRU8 accessed on 12 January 2024). The study design received approval from the Ethics Committee of the Faculty of Education and Sports Science at the University of Vigo (Code: 04-1421; Date of approval: 14 April 2021).

### 2.2. Measurements

#### 2.2.1. Energy Expenditure

Energy expenditure during activities was measured using a Fitbit wristband, a device that has been utilized in previous research [30]. This device incorporates accelerometers and optical plethysmography, recording data in 30 s epochs to track physical activity variables such as steps, energy expenditure, and active minutes. For the purposes of this study, the wristband was set to the “normal” mode.

To calculate the energy expenditure associated with physical activity, we used the MET formula: MET = 0.0175 kcal·kg^−1^·min^−1^. MET levels are divided into three categories: light activity (less than 3 METs), moderate activity (3 to 6 METs), and vigorous activity (greater than 6 METs) [31].

#### 2.2.2. Anthropometry

Body height was determined with a Stanley PowerLock flexometer, and body weight was examined using a Body Composition Monitor BF511 (Omron, Kioto, Japan). Weight (in kilograms) and height (in meters) were recorded with participants not wearing shoes and dressed in light clothing. Each child’s body mass index (BMI) was then calculated using the formula: BMI = body mass/height^2^.

### 2.3. Procedure

The research protocol entailed the measurement of energy expenditure among participants engaged in eight different after-school sporting activities, utilizing the Fitbit Charge 4TM (Fitbit, Inc.; San Francisco, CA, USA). Table 1 shows a list of the sport activities included in the analysis along with their respective samples. Prior to the commencement of the activities, a specific measurement day was designated. Each participant was equipped with a Fitbit bracelet and was thoroughly briefed on its functionality. The wristbands were activated at the beginning of the sports activity. When the sport activity was finished, the wristbands were deactivated, and the energy expenditure of each child was recorded. The assessments were strategically divided into two sessions, conducted within the same week. The collection of anthropometric data was systematically performed during the inaugural session of the study.

### 2.4. Statistical Analysis

Statistics were computed using the Statistical Package for the Social Sciences (SPSS v24, Armonk, NY, USA: IBM Corp.). The statistical evaluation consisted of a Kolmogorov–Smirnov test to examine the normal distribution of the data. Continuous variables were reported as mean ± standard deviation (SD) when normally distributed or as median and interquartile range (IQR) when not normally distributed, and categorical variables were presented as proportions. Kruskal–Wallis and Mann–Whitney U-tests were used to determine differences between groups. Additionally, effect sizes for pairwise comparisons were calculated using the formula r = z/√n [32]. They were interpreted as small (<0.3), moderate (0.3 to 0.5), or large (>0.5) [33]. The level of significance was set at a *p*-value less than 0.05.

## 3. Results

All participants completed the protocol and final testing; there were no dropouts. Furthermore, no adverse effects or complications were observed at any point during the course of the study. Consequently, a total of 129 healthy children (mean ± SD: age, 11.35 ± 0.55 years; height, 149 ± 8.8 cm; body weight, 42.89 ± 11.85 kg) were included in the analysis. The final sample consisted of 22.48% girls, indicating a nonbalanced representation of both genders (Table 2). Furthermore, certain sports such as soccer, futsal, or rugby were only practiced by boys in the sample included in this study.

Table 3 shows the results for energy expenditure, taking into account the sport modality practiced. Data analyses revealed significant differences between the eight sports in total calories per session, calories per minute, and METs (*p* < 0.05). Compared to chess, all sports showed higher energy expenditure (*p* < 0.05) and the effect sizes of the changes in comparison to chess were large (r > 0.50) in all groups. Soccer and rugby showed the highest energy expenditure per session. No significant differences were found between soccer and rugby at any measurement (*p* > 0.05), with a moderate effect size for total calories per session (r = 0.38), and a small effect size for calories per minute of activity and METs (r = 0.29 and 0.29, respectively). However, when analyzing expenditure per minute of activity, rugby and taekwondo showed the highest energy expenditure.

When analyzing the results, a distinct contrast was observed based on the nature of the sport, categorized as either individual or team sports (see Table 4). Team sports demonstrated significantly higher energy expenditure per session compared to individual sports (*p* < 0.01, r > 0.30). Additionally, in team sports, both the average calories burned per minute (*p* = 0.01, r > 0.40) and the METs (*p* < 0.01, r > 0.40) were significantly higher than those recorded in individual sports.

## 4. Discussion

The primary goal of this research endeavor was to thoroughly investigate the energy expenditure associated with after-school sports activities among primary school children. The findings obtained from this study offer valuable insights and guidance for parents, guardians, teachers, and professionals aiming to develop effective physical activity strategies for children. Furthermore, the data presented here are significant as they contribute to the scientific understanding of energy expenditure during childhood, providing a foundation for further research and application in educational and health-related contexts.

Promoting physical activity in children remains a priority for public health [34]. Several investigations have confirmed that school can be an ideal setting for increasing energy expenditure through the practice of physical activity and sports [35,36]. The results of our study suggest that participation in after-school sport activities significantly increases energy expenditure, with an average of 5–6 METs. Other researchers have found improvements in children energy expenditure through different exercise training programs [37,38], but to the very best of the authors’ knowledge, scant research exists regarding the impact on energy expenditure of after-school sport programs.

The differing levels of energy expenditure among various sports modalities underscore the significance of taking into account the specific characteristics of each activity. Previous research has indicated that sports such as basketball, soccer, jogging, or running are activities that result in the highest energy expenditure [39,40,41]. In our study, soccer and rugby were found to be particularly energy-intensive activities, as has also been observed both in the compendium of energy expenditure for youth [17] and for adults [42]. Particularly, rugby involves high-intensity running, which likely contributes to the increased energy expenditure associated with this sport [43]. These results are in line with previous studies suggesting that there is a strong association between participation in sports activities and higher levels of energy expenditure [44] and physical activity [45] in children. In this regard, it should also be noted that participating in sports activities is strongly associated with improved physical fitness in children [46], including endurance, strength, power, and agility [47].

The World Health Organization recommends practicing physical activity in the range of 3 to 6 METs [48]. All sports included were in line with this recommendation, with the exception of chess, which showed the lowest intensity. However, parents consider its practice to be very positive as it improves their children’s cognitive and emotional skills [49]. According to our findings, it appears advisable to supplement chess practice with another sport that demands higher energy expenditure. Conversely, sports such as soccer, taekwondo, and rugby demonstrated the highest intensity, each exceeding 6 METs. An interesting contrast was observed when the data were analyzed based on the classification of sports into individual or team activities. While the time differences per session were not statistically significant, team sports consistently showed substantially higher energy consumption compared to individual sports. This distinction highlights the varying demands and benefits associated with different types of sporting activities. These results are in agreement both with those reported by Thiel et al. [50], who indicated that team sports involve a higher energetic expenditure, and with the compendium of energy expenditure for youth [17]. Our findings align with previous research, indicating that practicing team sports leads to higher energy expenditure [51].

Another noteworthy finding from our study is that during individual sports sessions, children engaged in physical activity at moderate to vigorous intensity for 23.81% of the time. However, this percentage significantly increased to 45.13% during team sport sessions. Engaging in team sports such as soccer, handball, or rugby at sports schools during out-of-school hours appears to be an effective strategy to help meet the recommended minimum physical activity levels for children [17,52]. This suggests that team sports might offer a more dynamic and engaging environment that promotes higher levels of physical activity among children.

There is a significant prevalence of dropout from after-school sports activities during the school years in Spain [53]. This dropout, coupled with a subsequent decline in overall physical activity levels, becomes characteristic during adolescence, and it intensifies notably during the transition to adulthood, particularly among girls compared to boys [14,54]. It has been suggested that dissatisfaction among participants may be a contributing factor to this withdrawal from extracurricular activities [53]. Addressing this aspect could potentially be achieved through implementing a series of measures aimed at optimizing these activities.

Despite the novel results provides for this manuscript, it is essential to recognize some limitations. For instance, the nonrandomized design and small sample size of our study may constrain the generalizability of our findings. Conducting research with a larger sample size and employing more robust study designs would yield data that could be extrapolated to a broader population and compared across different settings, thus offering a more comprehensive understanding of the subject matter. Another limitation of our study is the limited number of sessions analyzed. It would be advisable for future research to analyze after-school sport activities over an entire season, incorporating long-term follow-up to enhance the comprehensiveness and reliability of the findings. This approach would provide a more detailed understanding of the impacts and benefits of sustained participation in these activities. Also, it is important to note that the study was conducted in a specific geographic region (in the north of Spain). Consequently, the results derived from this sample may not necessarily be representative of the broader general population due to potential regional variations in physical activity and sports participation.

## 5. Conclusions

This study significantly enhances our understanding of the patterns of energy expenditure through the characterization of after-school sports activities among primary school children. The findings reveal that, within this particular age group, engaging in team sports leads to a greater amount of energy expended compared to participation in individual sports. Soccer, futsal, and rugby are the sports programs that demand the highest energy expenditure. However, these programs are currently only accessible to boys. It is essential to provide girls with equal access and opportunities to participate in these sports to enhance their physical fitness and wellbeing. This observation emphasizes the critical importance of advocating for regular physical activity as a means to bolster children’s overall health and wellbeing. To further optimize the effectiveness of physical education programs, it is advisable for educators and health professionals to carefully consider and implement interventions that are specifically tailored to address gender disparities and the distinct types of sports activities.

## Figures and Tables

**Table 1:** Description of the activities analyzed.

	n (%)	Time/Session (min)	Effective Time/Session (min)
Chess	11 (8.53)	60	60.59
Athletics	24 (18.60)	75	64.62
Handball	25 (19.38)	60	44.2
Soccer	28 (21.71)	90	72.6
Futsal	10 (7.75)	75	58.45
Judo	10 (7.75)	60	45.40
Rugby	14 (10.85)	90	61.07
Taekwondo	7 (5.43)	60	48.07
Total	129		

**Table 2:** Demographic data categorized by gender (n = 129).

		Boys (n = 100)		Girls (n = 29)		*p*-Value
		n (%)	$\bar{x}$ ± SD	n (%)	$\bar{x}$ ± SD	
Age (years)			11.35 ± 0.54		11.35 ± 0.61	*p* = 0.908
Activity						
	Chess	7 (7)		4 (13.79)		
	Athletics	10 (10)		14 (48.28)		
	Handball	22 (22)		3 (10.34)		
	Soccer	28 (28)		0		
	Futsal	10 (10)		0		
	Judo	7 (7)		3 (10.34)		
	Rugby	14 (14)		0		
	Taekwondo	2 (2)		5 (17.24)		
Body mass (kg)			42.8 ± 12.42		43.22 ± 9.8	*p* = 0.488
Height (cm)			148 ± 89		150 ± 82	*p* = 0.176
BMI (kg/m^2^)			19.16 ± 3.78		18.93 ± 2.96	*p* = 0.791

Abbreviations: BMI: body mass index; SD = standard deviation; $\bar{x}$ = mean.

**Table 3:** Description of the energy expenditure in the different sports analyzed.

Measures	Activity	Median [IQR]	*p*-Value
Calories/session			<0.01
	Chess	74 [68.5] ^a,b,c,d,e,f,g^	
	Athletics	272.75 [65] ^a,h,i,j,k^	
	Handball	209.50 [112.3] ^b,h,l,m^	
	Soccer	326.75 [112.3] ^c,i,l,n,o,p^	
	Futsal	243.75 [56.1] ^d,n,q,r,s^	
	Judo	177 [33.3] ^e,j,o,q,t,u^	
	Rugby	311.50 [174.5] ^f,m,r,s,t^	
	Taekwondo	211.50 [34] ^g,k,p,u^	
Calories/minute			<0.01
	Chess	1.38 [0.72] ^a,b,c,d,e,f,g^	
	Athletics	3.87 [0.57] ^a,h,i,j^	
	Handball	4.29 [0.75] ^b,h,k,l,m^	
	Soccer	4.40 [0.77] ^c,i,n,o^	
	Futsal	3.76 [0.50] ^d,k,n,p,q^	
	Judo	3.66 [0.69] ^e,l,o,r^	
	Rugby	4.86 [1.19] ^f,j,m,p,r^	
	Taekwondo	4.47 [0.83] ^g,q^	
METs			<0.01
	Chess	1.93 [1.00] ^a,b,c,d,e,f,g^	
	Athletics	5.39 [0.79] ^a,h,i,j^	
	Handball	5.97 [1.05] ^b,h,k,l,m^	
	Soccer	6.13 [1.07] ^c,i,n,o^	
	Futsal	5.24 [0.70] ^d,k,n,p,q^	
	Judo	5.10 [0.96] ^e,l,o,r^	
	Rugby	6.78 [1.66] ^f,j,m,p,r^	
	Taekwondo	6.22 [1.16] ^g,q^	

Abbreviations: IQR = interquartile range; MET = metabolic equivalent of task. Two identical superscript letters in the “Median [IQR]” column indicates that those groups differ statistically significantly (at least *p* < 0.05) from each nether.

**Table 4:** Energy expenditure according to the modality: individual or team sport.

	Individual Sport (n = 53)	Team Sport (n = 76)	*p*-Value
	Median [IQR]	Median [IQR]	
Calories/session	211.50 [136.75]	257.75 [128.88]	<0.01
Calories/minute	3.82 [0.89]	4.40 [0.90]	0.01
METs	5.33 [1.24]	6.13 [1.26]	<0.01

Abbreviations: IQR = interquartile range; MET = metabolic equivalent of task.

## Data Availability

The original contributions presented in the study are included in the article; further inquiries can be directed to the corresponding author.
